# Efficiency of Intervention Counseling Program on the Enhanced Psychological Well-being and Reduced Post-traumatic Stress Disorder Symptoms Among Syrian Women Refugee Survivors

**DOI:** 10.2174/1745017902016010134

**Published:** 2020-07-30

**Authors:** Ahmad Sa’ad Saleh Alsheikh Ali

**Affiliations:** 1 Department of Psychology, The University of Jordan, Amman, Jordan

**Keywords:** Intervention program, Well-Being, PTSD, Refugees, Trauma, Civil war

## Abstract

**Background:**

The number of individuals displaced from their original countries due to civil wars, hunger, disasters, and international wars is increasing worldwide day by day. These refugees are more vulnerable to Post-Traumatic Stress Disorder (PTSD).

**Objective:**

The present study aimed to examine the effectiveness of the intervention program in improving the well-being and reducing Post-Traumatic Stress Disorder (PTSD) among a sample of Syrian refugee women in Jordan who had been displaced due to civil wars in Syria.

**Methods:**

The study recruited 40 Syrian refugee females in Jordan who were psychologically challenged, with high PTSD symptoms (assessed by PCL) and a psychological well-being impairment (assessed by PWD). The culture of Jordanian society discriminates and affects the Syrian refugee women, rendering them vulnerable to PTSD. Quasi-experimental design was used, wherein the participants were randomly distributed in experimental and control groups (n=20/group). The control group members did not receive any services related to psychological support or psychiatric medications, while the experimental group underwent a counseling program.

**Results:**

The present study demonstrated that the intervention program improves the well-being and reduces PTSD among the Syrian refugee women who constituted the experimental group. The intervention program and the PTSD manifestation were not affected by age. The present study recommended that the program be applied to the refugees in Jordan to improve the well-being of the women in the residential areas.

**Conclusion:**

Furthermore, an intervention on the Jordanian cultural impact on the refugee camps was also essential if the condition for the female refugees worsened. Lastly, the effect of Jordanian culture on Syrian refugees should be investigated since the literature presented a negative impact.

## 1 INTRODUCTION

Individuals displaced from their original countries due to civil wars, hunger, disasters, and international wars are refugees [1]. The number of refugees is increasing worldwide day by day.

Although the civil war began in Syria in 2011, it was officially declared by the UN on June 12, 2012 [2, 3]. Thus, millions of people sought refuge in neighboring nations such as Jordan. An individual is regarded as a Syrian refugee in Jordan if he/she arrived after March 15, 2011, due to the effects of the wars. Moreover, any Syrian citizen who had been in Jordan before March 15, 2011, but was unable to return due to the civil war is also regarded as a Syrian refugee [4]. According to the United Nations High Commissioner for Refugees (UNHCR), 97% of such individuals have registered as refugees [1].

Strikingly, the demographic characteristics of the Syrian refugees’ households in Jordan are alarming [5]. The average population of the families is young, with 45% aged < 15 years. The population constitutes more women than men aged > 25 years. About 22% of the households are headed by women, and 16% of all the households are single-parented [4], thereby deeming that the majority of the refugees are women and children.

The unfavorable conditions for the refugees make the women, who are subjected to sexual harassment, rape, forced marriages, early marriages, and constant divorce in Jordan due to the gender-insensitive culture, vulnerable [6]. The Jordanian culture is based on the traditional Arabic culture as well as Islamic laws, which makes the society patriarchal [7]. Additionally, Jordanian society doubles as a patrilineal society [8].

Meriwether (2018) also reported that in Jordan, the Syrian refugee women are affected by the division of labor; the women perform the household duties, primarily caring for the children, while the male is the breadwinner [6, 9, 10]. In case the Syrian women work outside, they are closely monitored by their male relatives [11]. These cultural practices are complicated for women refugees who are single parents in the camps. Additionally, assigning household duties to refugee women ensures that they are unable to provide for themselves, thereby leaving them at the mercy of men, who in turn, abuse them in marriages. Therefore, the culture in Jordan makes refugee women vulnerable to post-traumatic stress disorder (PTSD) [6].

Marriages for Syrian refugee women living in Irbid City are determined by Islamic laws and Arabic traditions [12]. Men are allowed to marry up to four wives, while women are expected to remain obedient to their husbands [13]. The Syrian refugee women can be married off by their fathers at the young age of 15 years [14, 15]. Upon marriage, a refugee woman becomes the property of the husband, who dictates every aspect of her life [16]. Additionally, tradition allows men to punish their wives as they please [12]. Also, the traditional Jordanian laws prohibit women from shouting or screaming for help, a practice that has worsened the situation for the Syrian refugee women living in Jordan [17].

Divorce is accepted but decided by the man and usually relies on the obedience index of the woman. Barlas (2019) argued that after divorce, the children are under the care of the father, while the woman is permitted to take care of them [13]. Furthermore, divorced refugee women are outcast in the Jordanian society [9]. Therefore, refugee women prefer to remain in abusive marriages either due to children or to protect their dignity in society. Thus, problems accrued from forced and abusive marriages cause mental challenges to the Syrian refugee women living in the Jordan society, which might later develop into PTSD.

A large number of refugees suffer from mental health disorders, such as PTSD, anxiety, and depression, as compared to the general population worldwide. However, it is prevalent in women due to war experiences and trauma before the migration. Moreover, women have PTSD due to post-migration stressors such as separation from families as well as the pressure of settling in a new country [18]. The situation sometimes becomes challenging during war experiences, which include torture, hostage, death of loved ones, sexual harassment, and physical violence [19]. Although the symptoms may last for months or years, exemplary self-care and time can heal the person [20]. Women are more attached to their children than men, and hence, they usually develop stress firstly due to the experiences that the children have to endure and secondly due to their own suffering.

The symptoms of PTSD may be displayed within a month in a similar event that might serve as the trigger, or the effects could be felt after several years. The symptoms, including intrusion, hyper-arousal, and avoidance, among women living in refugee camps, are distinct as the groups consist of a heterogeneous population. In some cases, irritability, fatigue, restlessness, and sleeping disorders are also associated with PTSD. These symptoms could affect the daily operation of an individual, such as relationships, work situations, and other social-related issues [19]. These women overreact, display aggressive mood and attitude swings, lose hope in the future, as well as, show a lack of interest in people and relationships. However, only limited studies are available on the prevention and intervention measures for PTSD. Therefore, the present study aimed to find the effectiveness of the intervention counseling program in enhancing the psychological well-being and reducing the PTSD symptoms among Syrian refugee women survivors. The hypotheses focusing on the mental health aspect were as follows:

• The well-being of the Syrian refugee women in the experimental group improves as compared to the control group after implementing the counseling program.

• There is no correlation between the overall well-being or sub-domains due to the counseling program and the participant’s age.

• The PTSD score in the experimental group decreases as compared to the control group after implementing the counseling program.

• There is no correlation between the level of PTSD due to the counseling program and the participant’s age.

## 2. METHODS

The present study followed the experimental approach by choosing a quasi-experimental design because the purpose the study aspires to screen the individuals who receive the highest scores in PTSD as measured by the study tool (PCL), in addition to those who experience deficiencies in mental health levels as measured by the study tool (PWD). This procedure prevents the possibility of random selection from members of the study community. Quasi-experimental design is usually chosen in such experimental conditions.

### 2.1 Participants

The present study recruited 40 Syrian refugee women in Jordan, aged between 30 and 50 years and identified as psychologically challenged. During the identification process in the in-home assessment, a screening criterion was applied randomly to the women residing in Irbid City to include the appropriate participants for the study. The cross-cutting tool by the American Psychiatric Association (2013) was used in the screening, which identified 56 women with the highest scores for psychological challenges [21]. However, after applying Ryff’s psychological well-being scales (PWB) and PTSD checklist (PCL), a total of 40 women, who scored the maximum in both instruments, comprised the final cohort and were randomly and equally assigned to the experimental and control groups [22-24]. During the period of the study, the control group did not receive any psychological support or psychiatric medications, while the experimental group underwent a counseling program.

### 2.2 Design and Tools

The PWB was used to measure the specific domains of psychological functioning, which were distributed into six dimensions: autonomy, positive relationships with others, environmental mastery, personal growth, the goal in life, and self-acceptance [22-24]. A quasi-experimental design was used with experimental and control group. The experimental group underwent counseling sessions, while the control did not receive any psychological support or psychiatric medications for comparative analysis. The PCL-Civilian version for Diagnostic and Statistical Manual of Mental Disorder 4^th^ Edition (DSM-IV) developed by Weathers & Litz (1994) consisted of 17 self-report items [25]. These items were aligned with the PTSD diagnostic criteria in DSM-VI [21]. Moreover, validity and reliability values indicated that the PTSD checklist (PCL) was suitable and reliable for the study based on its characteristics.

The counseling program for this study was developed based on the analysis of the survival of Syrian women. Three focus group meetings were conducted to obtain a general understanding of the main challenges faced by the participants in daily life while coping as refugees with PTSD. The program consisted of four primary domains, two sessions each. Both sessions targeted each domain and focused on sharing the stories between the participants, using psycho-education techniques to deal with the feelings related to each domain, and finding strategies that would help in coping with the challenges. The four basic domains were alienation, loss and grief, hope and hopelessness, and psychological problems. The program was implemented for 1.5 months, two sessions per week. However, two additional sessions were conducted for the opening and closing, respectively. Informed consent was obtained from the participants in the opening session, as well as for the pre-test, after conveying the regulations for the whole program. The closing session entailed a summary and post-test application. The counseling program was reviewed by experts and academicians in the field of Counseling and Clinical Psychology prior to implementation. The consulted parties modified both the content and procedures to suit the study.

### 2.3 Counseling Program Development

The group counseling program was designed based on a number research and literature efforts. Yalom (2005) has extracted number of fundamentals for group counseling which was adopted on the development of this program [26]. He has focused on the importance of group processes in the hope installation and socialization techniques. Moreover, feeling of commonality and belonging experiences was employed based on Jacobs, Masson and Harvill (2009) explanations [27].

After developing the first draft of the counseling program, it was shared with both professors in the field of counseling and clinical psychology and professionals who are working with traumatic refugees. This procedure aimed at obtaining their feedback about the program, some modifications were added to the program based on their feedback.

### 2.4 Procedure

The study targeted one of the local associations in Irbid City in Jordan that were dedicated to supporting the Syrian refugees via different psychosocial services. Permission was obtained from the Faculty of Arts Research Review Committee of The University of Jordan, and interested parties were informed before the program was conducted. The participants signed informed consent at the beginning of the study. The participation was voluntary, and the participants had the right to withdraw from the program at any stage of the cycle. Notably, the program was implemented by a clinical psychologist who was a licensed psychotherapist.

## 3 RESULTS

The efficiency of the intervention counseling program that improved mental health and reduced the PTSD symptoms among refugee Syrian women survivors was assessed. A total of 40 Syrian refugee women living in Irbid city in Jordan were recruited for the current study. The means and standard deviation for the overall well-being and sub-domains were calculated for both experimental and control groups (Table **1**).

The means among the experimental group increased in the post-test as compared to the control group. To test if this difference was statistically significant, a two-way MANOVA was conducted (Table **2**).

Additionally, the interactive means and standard deviation for the overall well-being and sub-domains were calculated for the counseling program and age (≤ 24 or ≥ 24 years) in both groups (Table **2**).

The means showed a difference between the experimental and control groups for both age groups. To test if this difference was statistically significant, a two-way MANOVA was conducted (Table **3**). The F-values for the counseling program of the overall well-being and the sub-domain are statistically significant at ≤ 0.05 with respect to the post-test for both experimental and control groups, respectively. The means showed that the overall well-being and sub-domains improved after the experimental group underwent the counseling program. Thus, this result supported the acceptance of the first hypothesis that the well-being of Syrian refugee women in the experimental group would improve as compared to the control group after implementing the counseling program.

Table **4** shows that the F-values for the interaction between the program and age for the overall well-being and the sub-domains are statistically significant at 0.05. This result supported the second hypothesis that there is no correlation between the overall well-being and sub-domains due to the counseling program and the participant’s age. Table **5** shows that the PTSD on the post-test in the experimental groups was increased as compared to the control group; however, the significant difference was estimated by two-way MANOVA.

Additionally, the means of PTSD on the post-test in the experiential group for ≥ 24 years age group also differed from the ≤ 24 years age groups (Table **5**). Thus, the interaction and the difference between the groups and ages were estimated by two-way MANOVA.

Table **5** shows that the F-value for the counseling program of the overall well-being is statistically significant at ≤ 0.05 based on the post-test for both experimental and control groups. This result supports the third hypothesis that the PTSD score in Syrian refugee women in the experimental group decreases as compared to the control group after implementing the counseling program. Furthermore, the F-value for the interaction between the program and the age with respect to the PTSD scale was also statistically significant at 0.05 (Table **5**). This result accepted the fourth hypothesis that there is no correlation between the level of PTSD due to the counseling program and the participant’s age.

## 4 DISCUSSION

The PTSD symptoms are classified into four categories: avoidance, adverse changes in the mood and thinking, intrusion memories, and changes in the emotional as well as physical reactions. The nature of symptoms differs among individuals and is subject to time [28]. Thus, we determined the efficiency of the counseling program, whether it would alleviate the PTSD symptoms among the female refugee survivors.

The data analysis (Tables **1** and **2**) revealed that the well-being aspects of the Syrian refugee women in the experimental group improved as compared to the control group after implementing the counseling program. Interestingly, the control group recorded nearly similar means in both pre- and post-test, while it was improved in all the domains in the experimental group. Nonetheless, the participants improved maximally in adjusting to the environment as compared to other domains. According to a study by Writers (2019), the environment construes the necessary awareness in an individual, thus becoming the most comfortable skill acquired by a person with a psychological disorder [29]. Other domains such as personal growth, autonomy, self- acceptance, positive relationships, and purpose in life are controlled by the hippocampus, amygdala, hypothalamus, and limbic cortex. During traumatic experiences, the amygdala stimulates the hypothalamus to produce the adrenaline hormone that influences the thinking, mood attitude, and physical response. PTSD causes this biological process to be systematic, thereby occurring frequently and with minimum stimulation. Although the other domains develop slower than environmental mastery, significant improvements are detected in all domains, confirming the first hypothesis.

The present study showed that age neither affects the counseling program nor the PTSD. The F-value of 0.01 (Table **4**) indicated that both age groups respond similarly to the intervention program. These findings were contradictory to those described in the study by Ditlevsen and Elklit (2010), which stated that age is a factor that determines the intervention program and the manifestation of PTSD among trauma victims [30]. However, the result was in agreement with another study, which showed that PTSD could occur at any age. The study argued that even children experience PTSD depending on their experiences [31].

The Syrian refugee women’s PTSD scores in the experimental group decreased as compared to the control group after the implementation of the counseling program (Table **5**). The score in the experimental group improved because the program worked by minimizing the psychological impact of their trauma, teaching them to enhance their personality traits, such as personal growth, self- acceptance, relation with others, and purpose in life. Also, resilience has been observed to cushion the stressors and assist towards a favorable outcome [32].

The survivors among the Syrian refugee women endure the impacts of traumatic memory. They experience distressing memories, which are recurrent and unwanted about the traumatic situation. The memory sometimes occurs in the form of flashbacks, making them relive the traumatic event as a fresh situation [33]. Occasionally, the intrusive memories manifest in the form of nightmares. These memories trigger emotional distress, which is occasionally coupled with physical violence against the person/object that is reminiscent of the traumatic event. The Syrian refugee women have had bitter experiences in both Syria and the camps, thus fueling the symptoms of intrusive memories [19].

Moreover, the avoidance symptoms include avoiding either thinking or talking about the traumatic situation and avoiding people and activities that remind about the traumatic event [20].

Therefore, the nature of symptoms experienced by the female refugees varies within the Irbid City due to the varied length of stay in the camps. McFarlane (2010) suggested that these women have a negative mood and thinking similar to that of a traumatized person [34]. The other prevalent condition is the feeling of hopelessness about the future. Some may also experience memory lapses, which would include not able to recall the significant aspects of the traumatic moment. Moreover, the refugee women in Irbid City feel detached from their family members and friends, thereby developing difficulty in maintaining relationships characterized by emotional numbness as well as lack of positive emotions [33]. The long-term effect of this condition has been realized to be affecting a large number of Syrian refugee women married in Irbid City.

These women show signs of overreaction and lack of tolerance due to lack of sleep and concentration and overwhelming feeling of guilt or shame; this coupled with irritability results in aggressive behaviors [33]. The negative emotions of guilt and shame are strongly correlated with maladaptive coping [35]. Such individuals feel easily threatened and are always vigilant that entail self-destructive behaviors, such as excessive use of drugs and driving extremely fast. Therefore, it can be deduced that the PTSD symptoms vary among the victims [31].

Intriguingly, the pre-migration stressors among the women in Jordan refugee camps include the loss of loved ones, family separation, loss of freedom, and challenging economic situations [28]. Before the war, some of the refugees had honorable jobs and compassionate families. These women were affected maximally during the pre-migration moments due to the abduction as well as the death of children. Females are more attached to children than males. LaFrance (2015) argued that during the gestation period, there are several activities within a woman’s brain sections, such as in the prefrontal cortex, parietal lobe, and the midbrain, which are responsible for social interaction, empathy, and anxiety [36]. The hormonal changes also develop protectiveness and overwhelming maternal love, which is controlled by the brain. Therefore, refugee women experience PTSD more than men under the same conditions.

However, another study showed that refugees who have experienced severe trauma are unlikely to develop PTSD. The researchers used two samples of participants constituting 50% refugees and 50% non-refugees. There is a need for immediate steps to provide protection to refuges to help recover psychological suffering arising from trauma and stress [37]. These findings stated that when the trauma is severe, it causes the suppression of the memories of the traumatic event. This phenomenon could be attributed to the dysfunctional gamma frequencies within the brain [38, 39].

However, the findings have certain limitations. The mental disorder comorbidities, such as anxiety or depression, which could significantly influence the PTSD and well-being in participants, were not assessed. The specific domains of psychological functioning were not rated by a clinician. Also, DSM-IV was used instead of DSM-V.

## CONCLUSION

The intervention program was designed for the Syrian refugee women living in Jordan. The experimental group showed an improved general score in the well-being as well as in the designated sub-domains (autonomy, environmental mastery, personal growth, positive relations, purpose in life, and self-acceptance). However, it was found that age does not affect the implementation of the program. The experimental group comprised of the PTSD sample selected scientifically from among the Syrian refugee women, thus making this program applicable. It could be concluded that the PTSD experienced by Syrian refugee women is triggered by the cultural practice of the Jordanian society that allows abusive marriages and discourages divorce while victimizing women even in the rape situations. The society is both patriarchal and gender insensitive. Therefore, additional studies on the Jordanian culture and its impact on the refugees, especially women living in Jordan, are imperative. The women should be sensitized to advocate for their rights within the refugee camps and avoid early marriages. The women should be taught remedies to fight rape, such as screaming that is discouraged by the Jordanian culture. Lastly, the current program was effective in the intervention of PTSD among the Syrian refugee women living in Irbid City and can be applied to help the majority who are suffering within the different cities of Jordan.

## Figures and Tables

**Table 1:** Means and standard deviation of the study groups in the Well-being scale domains.

**Dependent variable**	**Groups**	**Pre-test**			**Post-test**		
		**N**	**Mean**	**Std. Deviation**	**N**	**Mean**	**Std. Deviation**
Autonomy	Control	20	18.95	3.17	20	20.50	3.07
	Experiment	20	21.30	3.31	20	29.05	4.70
Environmental mastery	Control	20	20.65	4.06	20	19.60	3.60
	Experiment	20	19.85	3.39	20	32.15	5.05
Personal Growth	Control	20	19.80	3.56	20	21.05	3.36
	Experiment	20	20.55	2.84	20	28.25	3.64
Positive Relations	Control	20	20.10	3.14	20	20.65	3.13
	Experiment	20	20.45	3.75	20	28.35	3.92
Purpose in life	Control	20	19.65	3.54	20	20.45	3.62
	Experiment	20	20.80	3.99	20	28.40	4.31
Self-acceptance	Control	20	20.65	3.44	20	20.60	3.47
	Experiment	20	21.00	3.92	20	29.80	4.07
Overall well-being	Control	20	119.80	10.80	20	122.85	10.38
	Experiment	20	124.05	9.35	20	176.00	13.29

**Table 2:** Means and standard deviation of the well-being domains due to the interaction between Intervention and age.

	**Age (years)**	**Control group**			**Experimental group**		
		**Mean**	**Std. Deviation**	**N**	**Mean**	**Std. Deviation**	**N**
Autonomy	≤24	20.60	3.27	10	28.62	5.19	13
	≥24	20.40	3.03	10	29.86	3.85	7
Environmental mastery	≤24	17.20	2.10	10	30.46	5.44	13
	≥24	22.00	3.20	10	35.29	1.98	7
Personal Growth	≤24	21.20	2.94	10	27.46	2.60	13
	≥24	20.90	3.90	10	29.71	4.96	7
Positive Relations	≤24	19.30	2.87	10	28.31	3.47	13
	≥24	22.00	2.91	10	28.43	4.96	7
Purpose in life	≤24	19.10	4.01	10	26.62	3.01	13
	≥24	21.80	2.74	10	31.71	4.57	7
Self-acceptance	≤24	19.10	3.03	10	28.31	3.47	13
	≥24	22.10	3.35	10	32.57	3.82	7
Overall well-being	≤24	116.50	7.96	10	169.77	9.44	13
	≥24	129.20	8.64	10	187.57	11.84	7

**Table 3:** Two-way MANOVA analysis for well-being due to intervention, age, and interaction.

-	**Sources**	**Type III sum of squares**	**df**	**Mean square**	**F**	**Significance**
Autonomy	Pre-test autonomy	0.02	1	0.02	0.00	0.97
	Group	621.77	1	621.77	36.84	0.00
	Age	2.55	1	2.55	0.15	0.70
	Group × Age	4.70	1	4.70	0.28	0.60
	Error	590.71	35	16.88		
	Corrected total	1328.98	39			
Environmental mastery	Pre-test environmental mastery	22.00	1	22.00	1.58	0.22
	Group	1604.37	1	1604.37	115.01	0.00
	Age	229.45	1	229.45	16.45	0.00
	Group × Age	0.70	1	0.70	0.05	0.82
	Error	488.26	35	13.95	-	-
	Corrected total	2306.38	39	-	-	-
Personal growth	Pre-test personal growth	21.23	1	21.23	1.76	0.19
	Group	556.80	1	556.80	46.19	0.00
	Age	4.30	1	4.30	0.36	0.55
	Group × Age	16.49	1	16.49	1.37	0.25
	Error	421.93	35	12.06	-	-
	Corrected total	985.10	39	-	-	-
Positive relations	Pre-test positive relations	6.20	1	6.20	0.50	0.49
	Group	572.25	1	572.25	45.90	0.00
	Age	18.91	1	18.91	1.52	0.23
	Group × Age	17.52	1	17.52	1.41	0.24
	Error	436.38	35	12.47	-	-
	Corrected total	1072.00	39	-	-	-
Purpose in life	Pre-test purpose in life	5.79	1	5.79	0.46	0.50
	GROUP	725.32	1	725.32	57.54	0.00
	Age	149.71	1	149.71	11.88	0.00
	Group × Age	8.64	1	8.64	0.69	0.41
	Error	441.21	35	12.61	-	-
	Corrected total	1233.78	39	-	-	-
Self-acceptance	Pre-test self-acceptance	2.97	1	2.97	0.25	0.62
	Group	912.71	1	912.71	77.29	0.00
	Age	126.29	1	126.29	10.69	0.00
	Group × Age	3.09	1	3.09	0.26	0.61
	Error	413.32	35	11.81	-	-
	Corrected total	1390.40	39	-	-	-
Overall well-being	Pre-test overall well-being	23.13	1	23.13	0.26	0.61
	Group	28782.00	1	28782.00	321.74	0.00
	Age	2230.19	1	2230.19	24.93	0.00
	Group × Age	57.98	1	57.98	0.65	0.43
	Error	3130.99	35	89.46	-	-
	Corrected total	33651.78	39	-	-	-

**Table 4:** Means and standard deviation of the groups on the PTSD scale and the interaction between intervention and age.

Group	Pre-PTSD			Post-PTSD			≤24 years			≥24 years		
	N	Mean	Std. Deviation	N	Mean	Std. Deviation	N	Mean	Std. Deviation	N	Mean	Std. Deviation
Experimental	20	57.65	11.19	20	41.55	7.11	13	58.62	7.92	7	56.86	9.12
Control	20	59.45	8.01	20	58	8.16	10	42	7.15	10	41.1	7.43

**Table 5:** Two-way MANOVA analysis for PTSD due to intervention, age, and anteraction.

Source	Type III sum of squares	df	Mean square	F	Sig.
Pre-test PTSD	26.99	1	26.99	0.43	0.51
Group	417.11	1	417.11	6.69	0.01
Age	0.04	1	0.04	0.00	0.98
Group × Age	0.35	1	0.35	0.01	0.94
Error	2181.84	35	62.34		
Corrected total	4932.98	39			
